# Is educational attainment protective against developing dementia? A twin study of genetic and environmental contributions

**DOI:** 10.1007/s10654-025-01286-x

**Published:** 2025-08-12

**Authors:** Ellen E. Walters, Susan E. Luczak, Christopher R. Beam, Malin Ericsson, William S. Kremen, Robert F. Krueger, Kristian E. Markon, Matt McGue, Marianne Nygaard, Matthew S. Panizzon, Brenda L. Plassman, Chandra A. Reynolds, Perminder S. Sachdev, Anbu Thalamuthu, Keith E. Whitfield, Nancy L. Pedersen, Margaret Gatz

**Affiliations:** 1 Center for Economic and Social Research, University of Southern California, 635 Downey Way, Los Angeles, CA 90089-3332, USA; 2 Department of Psychology, University of Southern California, Los Angeles, CA 90089, USA; 3 Davis School of Gerontology, University of Southern California, Los Angeles, CA 90089, USA; 4 Department of Medical Epidemiology and Biostatistics, Karolinska Institutet, 171 77 Stockholm, Sweden; 5 Aging Research Center, Karolinska Institutet and Stockholm University, Stockholm 171 77, Sweden; 6 Department of Psychiatry, University of California, San Diego, La Jolla, CA 92093, USA; 7 Department of Psychology, University of Minnesota, Minneapolis, MN 55455, USA; 8 Department of Public Health, The Danish Twin Registry, University of Southern Denmark, Odense M DK-5230, Denmark; 9 Department of Psychiatry and Behavioral Sciences, Duke University, Durham, NC 27710, USA; 10 Institute for Behavior Genetics, Department of Psychology & Neuroscience, University of Colorado Boulder, Boulder, CO 80309, USA; 11 Centre for Healthy Brain Ageing (CHeBA), University of New South Wales, Sydney, NSW 2052, Australia; 12 Program for Research on Men’s Health, Hopkins Center for Health Disparities Research, Johns Hopkins University School of Public Health, Baltimore, MD 21205, USA

**Keywords:** Dementia, Education, Genetic correlation, Twin studies

## Abstract

Low educational attainment is recognized as a modifiable risk factor for dementia. Despite the commonly accepted notion that greater educational attainment confers lower dementia risk, few family-based studies have investigated the causal bases for the association. Using data from seven twin samples from Sweden, Denmark, Australia, and the US participating in the IGEMS (Interplay of Genes and Environment in Multiple Studies) consortium (*N* = 60,027, 10.92% with dementia), we tested whether twins who achieve higher education than their co-twins have lower risk of dementia. The primary analysis applied a multilevel between-within regression framework, supported by descriptive statistics of within-pair differences. Results confirmed an overall association between educational attainment and dementia risk, such that individuals with higher educational attainment had less likelihood of developing dementia (phenotypic regression coefficient = −0.68, *p* <.0001). Within twin pairs, however, twins who achieved greater education than their co-twins did not uniformly show lower dementia risk (within-family regression coefficient = −0.07, *p* =.0983, while between-family regression coefficient = −0.98, *p* <.0001). Taken together, the pattern of results shows that the effect of educational attainment on dementia risk is largely attributable to genetic influences in common to educational attainment and dementia, although there are also contributions from environmental influences shared between members of the same family. Results were similar in men and women. These findings add to the literature by using a co-twin control design to address possible reasons that low educational attainment is associated with greater dementia risk.

## Introduction

Low educational attainment is a recognized putatively modifiable risk factor for dementia. It is strongly featured in consensus documents about preventing onset of dementia [1, 2]. At the same time, not every study has confirmed a significant association between educational attainment and dementia. A systematic review found that 58% of included studies supported an association between lower education and dementia risk [3], and a subsequent meta-analysis of prospective cohort studies showed that 73% of included studies supported an education-dementia association [4]. Some studies [5, 6] suggest that higher educational attainment may have a stronger effect on lowering women’s dementia risk than men’s, while others have shown the reverse [7]. Moreover, mechanisms accounting for the education-dementia association remain unresolved.

One explanation is genetics. For example, it is possible that apolipoprotein E (*APOE*), the major genetic risk factor for AD, moderates the association. In one report, low educational attainment was significantly associated with higher dementia risk only in *APOE* ε4 carriers, or in those at greater genetic risk for AD as indicated by their polygenic scores, and not in non-*APOE* ε4 carriers or those at lower genetic risk for AD [8].

Secular changes in education and dementia have also been used to explain the education-dementia association. Observations of rates of dementia in various countries over time have noted decreases in dementia [9], with some showing that education partially accounted for reduced incidence of dementia over recent decades [10, 11]. The effect may further be more pronounced in men than in women [12].

Natural experiments are another source of evidence, comparing dementia risk in those experiencing an educational reform that increased the number of required years versus those whose education just preceded the reform. In Great Britain, the mandatory rise in school-leaving age was associated with higher cognitive scores [13]. Yet, the positive effects of educational attainment on cognitive ability may not transfer to effects on dementia risk. In Sweden, for example, a reform that increased compulsory years of schooling by one year had a negligible and statistically non-significant effect on dementia risk [14].

Livingston et al. [1] underscored the challenge discerning whether greater educational attainment in adulthood reflects the consequences of greater cognitive ability or an advantage above and beyond cognitive ability. In support of the former, Kremen et al. [15] showed that, once accounting for general cognitive ability measured at age 20, further attained education was no longer associated with cognitive functioning 40 years later. Others used sibling models to control for familial factors and showed that adolescent cognitive ability and higher educational attainment each contributed to protecting against poor late life cognition function [16].

Mendelian randomization designs, an approach that avoids the biasing effect of reverse causation, have been applied to disentangle the role of general cognitive ability in explaining the education-dementia association. One such study reported that genetic variants connected to greater educational attainment were significantly associated with reduced odds of AD, while genetic variants connected to higher intelligence were less robustly associated with reduced odds of AD [17]. Another group found a small genetic correlation between educational attainment and AD, and that this association was not explained by *APOE* [18]. In contrast, two studies using Mendelian randomization subsequently found that the genetic effect of intelligence on Alzheimer’s disease risk explained any genetic effect of educational attainment on AD risk [19, 20].

Twin studies of education and dementia offer a way to control for the confounding effects of genetic and shared environmental variance, i.e., familial influences, thereby strengthening the test of whether educational attainment has a causal influence on dementia. In a co-twin control design, twins from the same family serve as each other’s controls. Here the co-twin control design is operationalized by a mixed-effects (or multilevel) regression framework [21]. This framework decomposes effects of the exposure (e.g., education) on the outcome (e.g., dementia) into between-twin-pair and within-twin-pair terms [22]. If the outcome is caused by the exposure, outcome and exposure should be significantly associated at the within family level, i.e., within monozygotic (MZ) pairs of twins there should be statistically significant effects of lower educational attainment on greater dementia risk. The presence of genetic and shared environmental confounds is assessed by examining the patterns of attenuation in the parameter estimates for MZ and dizygotic (DZ) twins. The exposure-outcome association within twin pairs is termed “quasi-causal” to denote the observational nature of twin studies despite using twins as a natural experimental research design [22].

In the present study, we apply the co-twin control design within a large international consortium of twin studies to test the effects of educational attainment on likelihood of dementia. We also directly compare MZ twins, DZ twins, and unrelated pairs matched on birth year and sex who correspond to what would be found in a non-twin sample.

## Materials and methods

### Sample

All samples included in the analyses are part of the Interplay of Genes and Environment in Multiple Studies (IGEMS) consortium [23, 24]. We included seven samples from four countries (Denmark, Sweden, Australia, and the US), all of which are part of the IGEMS consortium. Each sample was required to have data on education as well as either clinical dementia diagnoses or scores on a latent dementia index (LDI) [25]. Participants were aged 50 to 108 years at the time of dementia diagnosis or time of the assessment used to create the LDI score. The combined analytic sample was 60,027 individuals.

The Danish sample was drawn from two studies of the Danish Twin Registry (DTR), the Middle Age Danish Twins Study (MADT) [26] and Longitudinal Study of Aging Danish Twins (LSADT) [27]. MADT is a longitudinal study of same and opposite-sex twin pairs born between 1931 and 1952 first assessed in 1998, resulting in an age range from 45 to 68 years. LSADT is a cohort-sequential study of same sex twin pairs that began in 1995 and included an age range from 70 to 96 years.

The Swedish sample was drawn from the Swedish Twin Register (STR), a population-based register of twins born in Sweden since 1886 [28]. The sample included individuals who participated in one or more of the following studies: Swedish Adoption/Twin Study of Aging (SATSA) [29], a longitudinal study that began in 1984 and includes same-sex twins who indicated they had been reared apart along with a matched sample of twins reared together, with assessments conducted through 2014; Origins of Variance in the Oldest-Old (OCTO-Twin) [30], a longitudinal study of same-sex twin pairs over the age of 80 at baseline in 1991 with assessments conducted through 2002; Ageing in Women and Men: A Longitudinal Study of Gender Differences in Health Behavior and Health among Elderly (GENDER) [31], a longitudinal study of opposite-sex twin pairs born between 1916 and 1925 and followed from 1995 to 2005; Screening Across the Lifespan of Twins Study (SALT) [28], a telephone interview study of all twins born before 1958 conducted between 1998 and 2002; and the Study of Dementia in Swedish Twins (HARMONY) [32], a study that conducted a clinical workup of all twins 65 + years old who screened positive for dementia in SALT along with their co-twin and a control sample of healthy twin pairs evaluated between 1998 and 2002.

The Australian sample was from the Older Australian Twins Study (OATS) [33, 34], a longitudinal study that began in 2006 with the recruitment of twins aged 65 + years, including twins from the Australian Twin Registry as well as volunteers from the community.

The remaining four samples were from US twin studies. The National Academy of Sciences-National Research Council Twin Registry (NAS-NRC) [35] is a longitudinal registry of approximately 16,000 White male twin pairs born between 1917 and 1927 with both twins having served in the military. Those still alive were recruited for a dementia study between 1990 and 2002 [36]. The Vietnam Era Twin Study of Aging (VETSA) [37] is a longitudinal study of a national sample of male twins who served in the military at some time during the Vietnam era (1965–1975) and were 51 to 61 years of age at initial assessment in 2003 through 2008. The Carolina African-American Twin Study of Aging (CAATSA) [38, 39] is a cross-sectional study of a population-representative sample of African American twins ranging in age from 20s to 80s living in North Carolina recruited between 1999 and 2003. The nationally-representative Midlife Development in the United States (MIDUS) [40] study includes a twin subsample supplemented through snowball recruitment [41], with individuals of ages ranging from 34 to 82 years assessed by telephone in 2004 to 2006 and in 2013 to 2014.

## Variables

### Education

Harmonization of educational attainment was performed in all IGEMS samples based on the International Standard Classification of Education (ISCED) that uses nine categories ranging from less than primary education through graduate degree [42]. In the phenotypic analyses, all nine categories were retained. In the genetic models, the categories were collapsed into three categories to avoid overfitting with 1 = those with lower secondary education or less (categories 0–2), 2 = those with upper secondary, post-secondary or short cycle tertiary (categories 3–5), and 3 = those with a bachelor’s degree or higher (categories 6–8).

### Biological sex

Sex assigned at birth was coded female = 1 and male = 0.

### Race

Race and ethnicity were assigned using standard U.S. Census categories in the CAATSA, VETSA and MIDUS samples using self-report data. All participants in the Swedish, Danish, Australian and NAS-NRC samples were coded non-Hispanic white. Race was coded Black = 1 and non-Black = 0.

### Zygosity

Zygosity was originally assigned based on questions about intrapair similarities in childhood, with this method validated repeatedly with DNA as having 95–98% accuracy [43–45]. All participants seen in person have been invited to provide DNA, and all twin pairs with genotyping have had their zygosity confirmed. (coded dizygotic, DZ, = 1, monozygotic, MZ, = 0).

### Dementia

Dementia was determined in one of two ways in each study, either by a diagnosis (via a clinical workup by study personnel or a diagnosis recorded in a national registry), if available, or by a cut-off score on the LDI [25] if no clinical diagnosis was available. Individuals with LDI scores below the threshold corresponding to a dementia diagnosis were categorized as not demented. (coded dementia = 1, no dementia = 0).

*Clinical dementia diagnoses* were available in three of the samples, STR, OATS, and NAS-NRC, for everyone aged 65 years and older. OATS used a one-stage procedure with a complete clinical workup. STR and NAS-NRC used a two-stage protocol [46–48] with diagnoses assigned at multidisciplinary consensus conferences according to the current diagnostic and statistical manual (DSM) criteria for dementia [49].

In the STR, all individuals, including those who did not receive a clinical workup (due to being too young at the time) were also followed through linkage with national registries through 2016 or 2017, where dementia diagnostic codes from the current ICD [50] were available. Prior comparison of the registries to clinical diagnoses indicates sensitivity of 55% and a specificity of 98% [51, 52]. The low sensitivity for registry diagnoses means some cases will be misclassified as not demented, which will increase chance of type II error.

*Latent Dementia Index* (LDI) scores were used for four of the samples, DTR, CAATSA, MIDUS, and VETSA, which had test batteries assessing cognitive ability, memory, and functional daily living skills, but did not have clinical dementia diagnoses. The LDI is a tool for estimating dementia risk through its incorporation of cognitive and memory performance with functional ability (i.e., instrumental activities of daily living) net of general cognitive ability [25]. LDI scores were also calculated for OATS and for STR participants seen in person. Only the NAS-NRC sample does not have any LDI scores. Lower LDI scores indicate greater likelihood of dementia.

In previous work with the IGEMS consortium, we showed that the LDI is heritable and has considerable genetic overlap with clinically-based dementia diagnoses. We used the LDI as a continuous measure of dementia likelihood, but also as a categorical variable by identifying cut-off scores along the LDI distributions in each study. We previously derived cut-off scores by first validating LDI against clinical diagnoses in studies with both diagnoses and LDI, then applying the procedure to studies without clinical diagnoses. We then aligned the cut-off scores across studies to distinguish individuals with and without probable dementia. In the between-within regression models we used the LDI cut-off variable to indicate dementia.

### Age

For those diagnosed with dementia, age is age of onset, i.e., age at which they met the threshold for dementia criteria. For those whose dementia was ascertained from the national registries in the Swedish Twin Registry, age was adjusted by subtracting 5 years from the registry age for those ascertained by the National Patient Register (NPR), the Outpatient Register (OPR), or the Prescribed Drug Register (PDR) and subtracting 7 years for those ascertained by the Cause of Death Register (CDR), consistent with prior validation work [51, 52].

For those in NAS-NRC and OATS who were clinically assessed but not diagnosed as demented, the age used was “age at last follow-up.” For those in the Swedish Twin Registry, the age used was “age at their last time of in-person assessment” or, for those with registry information, the earlier of December 31, 2016, or age at death.

For LDI, age was their age at the last in-person assessment when tested on cognitive, memory, and functional activity; or, for those subsequently reviewed for clinical diagnosis, the assessment corresponding to onset of dementia.

Given the small number of individuals above 90 years, for model testing we reclassified all participants over 90 years old as 90 years.

### Data analyses

The first step was the descriptive results of the individual IGEMS samples as well as results aggregated across all samples.

The second step was identifying pairs who were discrepant on educational attainment to look at intrapair differences on dementia likelihood. These included MZ pairs, DZ pairs, and a sample of unrelated pairs. Pairs were considered discrepant on educational attainment if they did not have the same ISCED category on the nine-category classification scheme. There was a total of 5058 twin pairs where both members of the pair had known ISCED and LDI scores as well as known zygosity. In all, 2,448 of these twin pairs (or 48.4%) were discrepant on educational attainment.

The sample of unrelated pairs was constructed by randomly assigning each participant with LDI scores to an unrelated participant matched on sex and year of birth, drawn without replacement. The result was 6,513 unrelated pairs discrepant on educational attainment. Pairs not discrepant on educational attainment (*N* = 3447) and individuals for whom matches were unavailable (*N* = 863) were excluded from the analysis. We then calculated the intrapair difference in continuous LDI scores by sex within MZ pairs, DZ pairs, and unrelated matched pairs, taking the LDI score of the member with the higher ISCED minus the LDI score of the member with the lower ISCED, and showing the result as a matched t-test.

The third step was the primary set of analyses using a co-twin control design. Twins from the same family serve as each other’s controls: intrapair analyses of MZ twins fully control for genetic and shared environmental confounding (i.e., any nongenetic factor that makes twins more likely to attain the same education status and dementia risk), while intrapair analyses of DZ twins control for one-half their genetic risk and all shared environment. Both MZ and DZ twin designs control for environments shared by twins within the same family. Here, we applied a between-within regression model to test the hypothesis that twins whose education was higher than their co-twins had lower likelihoods of being diagnosed with dementia. The model, through taking into account which individuals are twins from the same family, as well as the zygosity of those twin pairs, statistically adjusts for within family effects, that is, the variance explained by unmeasured familial genetic and shared environmental factors that make twins alike [29]. The full model is:

$$[\text{Dx}]\_\text{ij}=e^{\wedge }\left(\pi \_0j+\pi \_1\;[\text{Educ}]\_j+\pi \_2\;[\text{Educ}]\_\text{ij}+\pi \_3\;[\text{Zyg}]\_j\right.\;+\pi \_4\;[\text{Educ}]\_\text{ij}\;\text{X}\;[\text{Zyg}]\text{\_}j+\pi \_5\;[\text{Female}]\_\text{ij}\;+\pi \_6\left[[\text{Female}]\_\text{ij}\;\text{XEduc}\right]\_\text{ij}+\pi \_7\;[\text{Female}]\;\_\text{ij}\;X\;[\text{Zyg}]\_j\left.\;+\pi \_8\;[\text{Female}]\_\text{ij}\;X\;[\text{Zyg}]\_j\;X\;[\text{Educ}]\_\text{ij}+\pi \_9\;[\text{Covs}]\_\text{ij}\right)$$

π_0j=γ_00+u_0j

Dementia diagnosis for person *i* in pair *j*, Dx_ij_, is in log-linear units. The parameter π_0_ is a random intercept that consists of the sample grand mean, γ_00_, and a deviation score of each twin pair, u_0j_, that takes into account within-family clustering to provide unbiased standard errors. The u_0j_ term constitutes the residual of the model (i.e., the best linear unbiased predictors). The fixed effect π_1_ is the effect of between-pair differences in educational attainment on twins’ dementia diagnosis. Conversely, π_2_ is the effect of within-pair differences in educational attainment on dementia diagnosis and tests whether twins with higher educational attainment have lower dementia likelihood scores than their co-twins. In MZ twins, π_2_ tests whether within-family confounds are environmental (i.e., “quasi-causal”) whereas in DZ twins the parameter also includes genetic confounds. The main effect of zygosity, π_3_, quantifies whether genetic variance accounts for dementia diagnosis statistically adjusting for between- and within-pair effects of educational attainment as well as all other covariates. Zygosity is necessarily included in the model as a lower-order term for purposes of testing whether within-family confounds are genetic or shared environmental in nature but is not of substantive interest. The effect of the interaction between education level and zygosity, π_4_, tests whether within-family confounds are genetic or shared environmental in nature. Thus, a statistically significant effect of π_4_ suggests that the within-family effect of educational attainment on dementia diagnosis is larger in DZ twins because of additive genetic confounds; null effects of the interaction suggest that the association between educational attainment and dementia diagnosis are shared environmental in origin. With the interaction between educational attainment and zygosity included in the model, π_4_ is the within-family effect of educational attainment on dementia diagnosis in MZ twins alone and represents the nonshared environmental regression effect. Parameters π_5_–π_9_ represent effects of covariates included in the model at the between (e.g., IGEMS study) and within-family (e.g., years of education) levels of the model. Finally, ε_ij_ is the residual variance of dementia and is estimated differently in the MZ and DZ groups.

All models were fit in SAS 9.4 [53] using PROC GLIM-MIX. We fit five models. The baseline phenotypic model included only twins’ three-category education scores (conceptually, the summed effect of parameters π_1_ and π_2_) in the model and tests the total phenotypic effect of education level on dementia. The second model includes both between-pair effects of educational attainment (π_1_) and within-pair effects of educational attainment (π_2_) in the model. A statistically significant π_1_ parameter suggests that familial confounds, including genetic, shared environmental, or both, account for the association between educational attainment and dementia. The third model included the main effect of zygosity (π_3_) and the effect of the interaction between educational attainment and zygosity (π_4_). The interaction effect tests whether the within-family effect of educational attainment on dementia is confounded by genetic variance (i.e., a statistically significant π_4_ effect) or shared environmental variance (i.e., π_4_ is not statistically significant). The fourth model includes a main effect for Female, while the fifth model includes all interaction terms with Female. As all interactions that included focal predictors were non-significant, we do not consider the fifth model further but present Model 4 as the final and most parsimonious model. Covariates of age and sample are included in all models.

A supplementary analysis applied the same modelling approach to Black twins only. Because of the reduced sample size, it was possible only to compute the first three models.

## Results

**Fig. 1:**
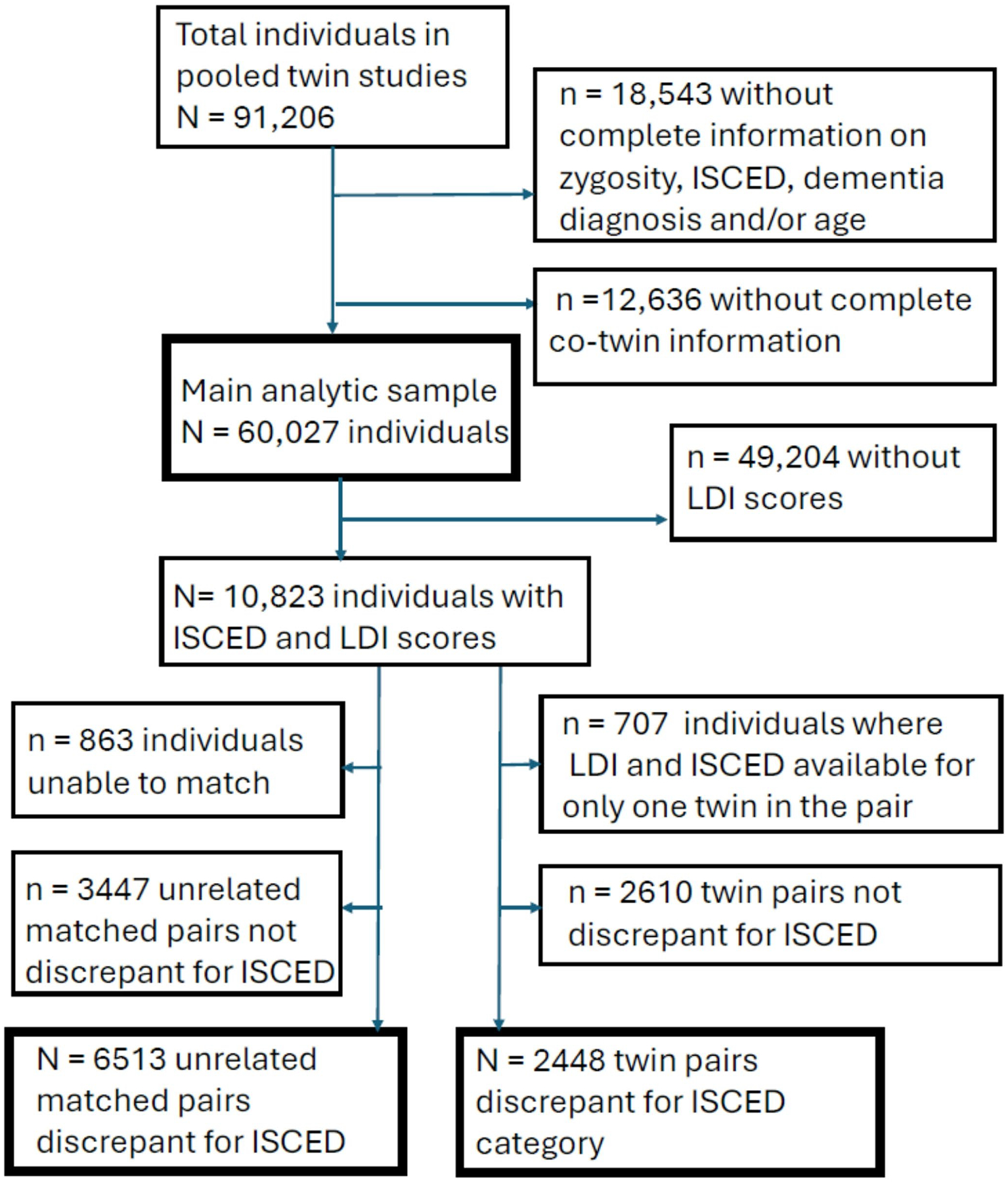
Participant flowchart. *ISCED* = International Standard Classification of Education. Unrelated matched pairs were matched for sex and birthyear. For those 18,543 missing information: n = 311 without zygosity, ISCED, dementia status, and age; n = 587 without zygosity and ISCED; n = 115 without zygosity, dementia status, and age; n = 3 without zygosity and age; n = 1032 without zygosity; n = 6172 without ISCED, dementia status, and age; n = 6 without ISCED and age; n = 3767 without ISCED; n = 5408 without dementia status and age; n = 1142 without age

Figure 1 provides a flowchart for sample derivation. Table 1 shows the descriptive statistics for the individual samples as well as aggregated across all samples. The total analytic sample of 60,027 was comprised of both members of 29,869 twin pairs as well as 289 singletons where complete pair information was known. There are 4123 female MZ (13.8%), 5317 male MZ (17.8%), 5973 female DZ (20.0%), 6503 male DZ (21.8%), and 7953 opposite-sex DZ pairs (26.6%). Of the 289 singletons, 68 are MZ, 132 are same-sex DZ, and 89 are opposite-sex DZ.

In the total analytic sample, 6252 (10.92%) were diagnosed or designated as having dementia based on clinical workup or a cut-off score on the LDI. In samples with longitudinal information, mean follow-up from the first wave of data collection to dementia onset, according to either clinical diagnosis or LDI cutoff, was 11.72 years (SD = 14.51 years).

**Fig. 2:**
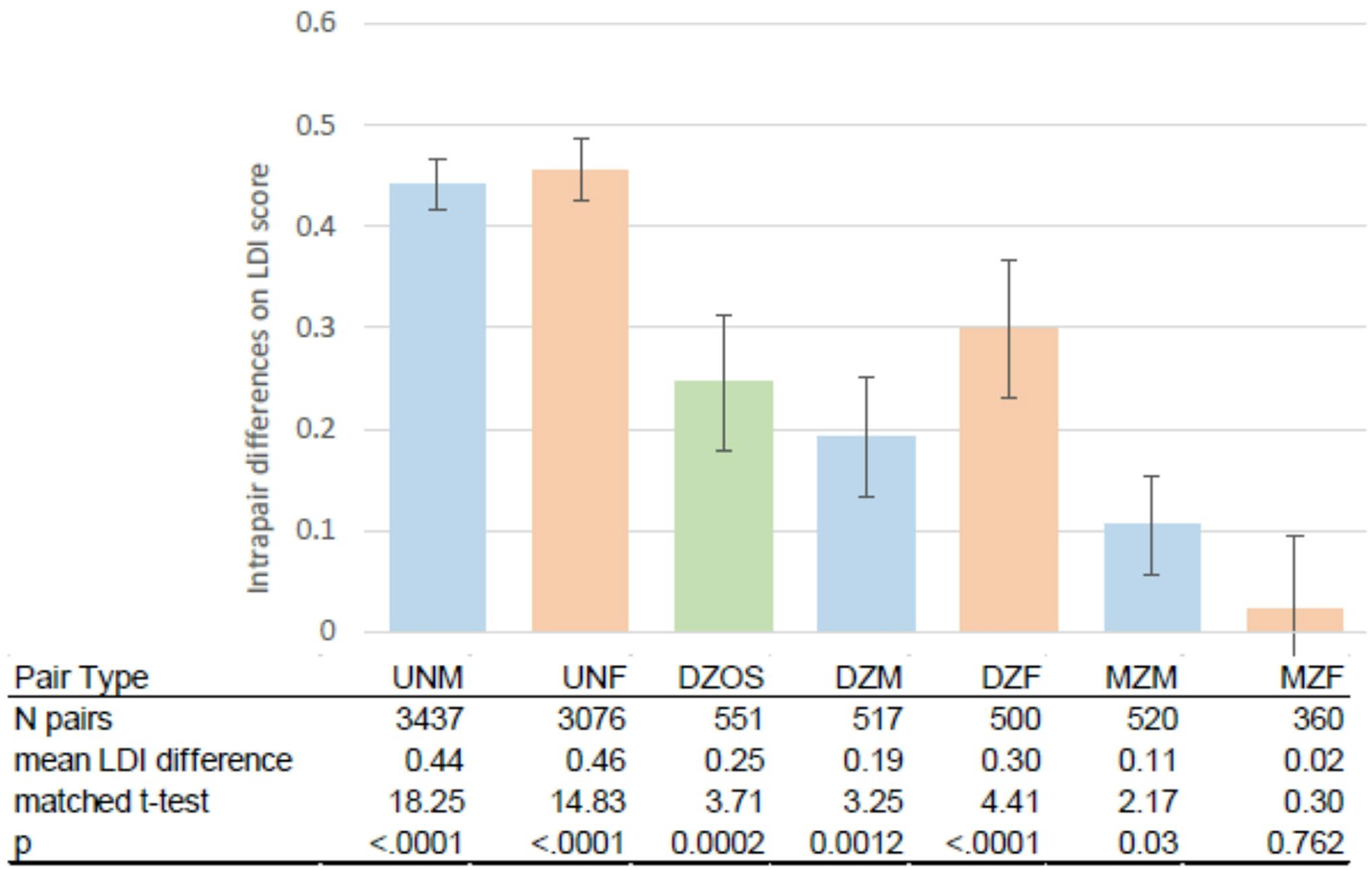
Mean difference in dementia likelihood within pairs who are discrepant for educational attainment stratified by genetic relationship and sex. *LDI* latent dementia indicator, *ISCED* International Standard Classification of Education, *UNM* unrelated male matched pairs, *UNF* unrelated female matched pairs, *DZOS* opposite sex dizygotic twin pairs, *DZM* male same sex dizygotic twin pairs, *DZF* female same sex dizygotic twin pairs, *MZM* male monozygotic twin pairs, *MZF* female monozygotic twin pairs. Blue columns indicate male pairs, peach columns indicate female pairs, the green column indicates opposite sex pairs. The bars are standard errors. The matched t-test compares the LDI scores of the member of the pair with the higher attained education to the member with lower attained education

Figure 2 displays within pair mean differences on likelihood of dementia (as measured by the LDI score) for pairs discrepant on educational attainment, i.e., they did not have the same ISCED category. These include twin pairs stratified by zygosity and sex, and the sample of unrelated pairs constructed from individuals who were matched on year of birth and sex. Except for MZ female pairs (where *p* =.762), the member of the pair with the higher education consistently had a lower likelihood of dementia (*p* <.0001 to *p* =.03). However, this difference varied across pair type. For both male and female pairs, the largest differences in dementia likelihood were seen in unrelated pairs, followed by DZ pairs, and then MZ pairs. For men, but not women, DZ twins did not have a significantly larger LDI difference than MZ twins, although both twin types had a smaller difference than the unrelated pairs.

Table 2 shows results of the co-twin control analyses using between-within regression. In all models, covariates include age (centered at 60 years) and sample. In Model 1, the bivariate regression shows a negative correlation between the total effect of greater educational attainment and dementia (π), beta = −0.68 (se = 0.03), *p* <.0001. In Model 2 with the total effect of education on dementia parsed into between-family (π_1_) and within-family (π_2_) effects, there were significant between-family but not within-family results, between beta = −0.98 (se = 0.03), *p* <.0001; within beta = −0.07 (se = 0.04), *p* =.0983. In Model 3, we find no significant main effects of zygosity (π_3_), beta = −0.02 (se = 0.03), *p* =.6393, but a significant interaction term between zygosity and within-family deviation scores on educational attainment (π_4_), beta = −0.26 (se = 0.10), *p* =.0101. In Model 4, we find a main effect of female (π_5_), beta = 0.33 (se = 0.03), *p* < 0001. Two- and three-way interactions of Female with Sample, Zygosity, and Education in Model 5 were largely non-significant and did not alter the conclusions indicated by the earlier models. Thus, Model 4 represents the most parsimonious solution. Supplementary materials available online include Table S1 showing Model 5 and all effects and interactions evaluated.

Supplementary Table S2 shows modelling results for the Black twins in the sample (*N* = 327). Model 2 contains both significant between- and significant within-family effects. A separate analysis with non-Hispanic white only was no different from the results for the total analytic sample. This result bears replication with a larger sample of Black twins.

A further supplementary analysis (Table S3) considered whether other variables known to affect dementia risk, specifically, body mass index, hypertension, coronary artery disease, stroke, physical activity, and social activity, modified the phenotypic association between educational attainment and dementia likelihood. This set of variables was available for only a subset of individuals. Results showed only small reductions in the regression coefficients for education once the covariate was included.

A sensitivity analysis (shown in Table S4) within each sample identified all twin pairs discordant for dementia where the co-twin control lived beyond the age of onset in the twin with dementia and never developed dementia. The frequency of pairs discordant for dementia where the twin with dementia had the higher education was compared to the frequency of pairs discordant for dementia where the co-twin control had the higher education. A significant difference in frequencies for MZ pairs would support a quasi-causal interpretation of the association between educational attainment and dementia risk. Attenuated results for MZ compared to DZ twins would support the interpretation that shared family environment plays a role in the education-dementia association. In the four separate samples with sufficient pairs to perform a statistical test, only for the DZ pairs in the DTR sample was there a significant difference where the twin with higher education was less likely to develop dementia than the twin with lower education. No significant findings were seen in any other sample. The analysis was repeated with the STR sample adding pairs where the co-twin control subsequently developed dementia, but at least 5 years later than the age of onset of the first twin with dementia. This analysis tests whether education might delay onset. Results were non-significant.

## Discussion

We undertook a test of mechanisms for the association between educational attainment and dementia risk; specifically, whether it can be considered quasi-causal or whether it is explained by genetic influences in common to educational attainment and dementia risk and/or environmental influences shared between twins in the same family. We confirmed the observation of a baseline association between lower education and greater dementia risk. Comparison of LDI scores within twin pairs to LDI scores within unrelated matched pairs discrepant on educational attainment shows a strong association between lower education and dementia likelihood in the unrelated pairs. The magnitude of the difference in education between individuals who did or did not develop dementia appears comparable to existing literature, although there is great heterogeneity in previously reported results [3, 4].

Using the between-within regression framework, we found that individuals with lower educational attainment are more likely to develop dementia, consistent with unrelated matched pairs. However, there was no statistically significant effect of educational attainment on dementia risk once controlling for familial confounding, i.e., genetic influences in common to educational attainment and dementia risk and environmental influences shared between members of a family. A significant between-family effect indicated that twins, regardless of zygosity, who come from families where the two siblings on average have higher educational attainment both have a lower risk for dementia. The non-significant effect of twins’ educational attainment on dementia within families indicated that in families where twins differed on education, the co-twin with the lower education was not necessarily likely to have a higher risk of dementia. Zygosity was included in the model in order to ascertain whether these familial confounds were genetic and/or shared environmental. Here, significant interactions for MZ and DZ twins in the association of educational attainment with dementia risk indicated that the familial confounding was largely explained by additive genetic variance in common to the exposure and the outcome.

The role of genetic influences was also evident in the comparison of continuous LDI scores for pairs discrepant on educational attainment. The smaller, though non-zero, difference in LDI scores for twin pairs compared to unrelated matched pairs illustrates substantial familial confounding, as shown in the between-within regression results. Further, the weaker results for MZ compared to DZ pairs supports interpreting the familial effects as genetic confounding. For men, however, the difference between MZ and DZ pairs was muted, possibly pointing to some role for environmental influences shared within a family (e.g., nutrition, intellectual stimulation).

To contextualize within-pair differences in educational attainment, for unrelated pairs, the difference was approximately 1.5 ISCED categories; for DZ pairs, one category; for MZ pairs, approximately three-quarters of a category. See Figure S1 in the supplementary material.

The pattern we observed with the co-twin control analyses is further supported by an earlier report from the Duke Twins Study of Memory in Aging in the NAS-NRC Twin Registry. Therein, Potter et al. [54] reported that in co-twin control models education was not a significant predictor of dementia, although education was a significant predictor in case-control models where cases and controls were not genetically related.

Our results on genetic confounding complement prior genetic studies. These include findings that genetic variants associated with greater educational attainment were associated with reduced risk of AD [17] and that there was a genetic correlation between educational attainment and AD [18]. Mendelian randomization results further indicate not only a genetic effect of educational attainment on AD risk, but also this effect was explained by the genetic effect of intelligence [19, 20]. This pattern would provide support for the argument of Kremen et al. [15] that it is most likely that general cognitive ability leads to both greater educational attainment and reduced dementia risk. Earlier, using data from SATSA, Pedersen et al. [55] found that the correlation between educational attainment and mental status score was primarily attributable to genetic effects for cognitive abilities; dementia diagnoses were not then available.

Previously, we looked at educational attainment and dementia in two cohorts from the Swedish Twin Registry [56] and found that twins who developed dementia were at least twice as likely to have lower education than their co-twins who did not develop dementia, although the difference was not statistically significant. In a larger sample from the Swedish Twin Registry, within MZ pairs only, we found the twin who developed dementia was three times as likely to have lower education than their co-twin who did not develop dementia, a difference that was statistically significant [57]. Most typically, however, twins in a pair had the same number of years of education, and on average twins who developed dementia had 0.85 fewer years of education than their co-twins who did not develop dementia [57]. In the present study, there was also a tendency toward the twin who developed dementia to have the lower education of the pair. At the same time, differences in the range of 0.85 years of education may be too small to be captured by differences between ISCED categories, and may not be educationally meaningful.

Unlike some prior reports [6], we found no interactions with sex. The significant main effect for sex likely reflects that the proportion of dementia cases is greater in women than in men in this sample. In turn, this difference is known at least in part to reflect the effect of women’s greater longevity on prevalence rates.

Limitations of the present study include the use of different outcome variables (e.g., DSM vs. ICD criteria for diagnosing dementia, clinical diagnoses vs. LDI) for different samples included in the pooled analyses. Although the LDI cutoff has been validated against dementia diagnoses [25], there are certainly misclassification errors. In the validation analyses, false positives were low (range: 1.7–7.0%) [25]. The performance of the LDI in mapping on to dementia diagnoses is superior to a cognitive composite that does not privilege memory impairment or include impairment in functional abilities [25]. Another limitation is that, although we control for age, we do not fully account for age differences in onset of dementia. The sensitivity analyses, however, show that incorporating relative survival of twins in pairs discordant for dementia supports the same conclusions.

A further limitation of the present study is the use of a harmonized measure of education, the ISCED. The advantage of the ISCED is that it was specifically developed to enable cross-country comparisons. However, the ISCED categories may not capture more subtle differences in educational attainment between twins in a pair, specifically differences of a year or two of education when the difference did not result in graduation from one level of schooling to another. Regardless, unrelated pairs had far greater within pair differences in education than did twin pairs.

Co-twin control designs have known limitations. Where within-pair twin correlations for the exposure are high, there is a risk that measurement error in the exposure will result in inflated associations between the family mean of the exposure and the outcome, and hence erroneous conclusions about the extent of familial confounding [58]. Although the exposure-outcome association is presumed causal or “quasi-causal”, the design remains observational and cannot completely rule out the possibilities of reverse causation and unique environmental influences on exposure in addition to actual causal effects.

Limitations with respect to generalizability of results should also be noted. The analyses are conducted with twins, leading to concerns with selection bias. Volunteer bias is minimized by the fact that the bulk of participants are from population-based twin registries with high levels of participation, while also including information from linkage to administrative records. Each of the twin studies has made some efforts to compare their samples to their respective national populations and found the twins to be similar to the population in general [37, 59, 60]. The phenotypic results describing the education-dementia association are similar to results from non-twin samples. We note that the samples are largely northern European in origin, from high-income countries. At the same time, when the older Scandinavian twins received their education, these countries would not have been recognized as high income. Similar analyses would be useful with samples from countries currently low- and middle-income. We did separately consider the African American participants available in the U.S. twin samples, with those results suggesting less genetic confounding and the possibility of some quasi-causal relationship between educational attainment and dementia in this sample, although this conclusion must be tentative due to sample size.

## Conclusion

By using a twin design with 60,027 participants from an international consortium, of whom 10.92% were determined to have developed dementia, we contribute to the question of what mechanisms explain the association between lower educational attainment and greater dementia risk. Twins within a family were highly similar in their educational attainment. To the extent that there were differences, the twin with the higher educational attainment did not reliably accrue greater protection against developing dementia. Findings from the co-twin control regression analyses supported the interpretation that the association between lower educational attainment and greater dementia risk is predominantly—but not completely—genetically mediated. Differences between families in education, not differences within families, are the significant driver of dementia risk. This conclusion is not to discount the importance of education, nor to discourage efforts to improve education and educational opportunities, but rather to say that reducing dementia risk by adding years of education is far from a straightforward causal path.

## Supplementary Material

Supplementary Material

**Supplementary Information** The online version contains supplementary material available at https://doi.org/10.1007/s10654-025-01286-x

## Figures and Tables

**Table 1:** Sample characteristics in each sample and in total analytic sample

		Zygosity			Age	Education	Female	LDI	Dementia
	N	MZ	DZ(SS)	DZ(OS)	M(SD)	M(SD)	%	M(SD)	%
DTR	5208	1956	2196	1056	67.0(11.56)	3.2(1.58)	54	6.28(0.76)	8.5
STR	44,765	11,822	18,332	14,611	73.6(9.76)	2.5(1.73)	55	6.55(2.06)	12.5
OATS	556	314	154	88	74.7(5.73)	3.9(1.66)	65	7.72(1.32)	4.5
CAATSA	234	88	98	48	59.5(8.53)	4.0(1.91)	59	6.85(1.19)	5.1
MIDUS	780	318	270	192	65.1(9.36)	4.9(1.54)	56	6.85(1.20)	8.5
NASNRC	7294	3748	3546	0	74.7(4.48)	4.6(1.78)	0	---	5.7
VETSA	1190	702	488	0	61.0(2.66)	4.6(1.36)	0	6.59(0.49)	0.3
Total	60,027	18,948	25,084	15,995	72.7(9.75)	2.9(1.88)	47	6.51(1.33)	10.92

*DTR* Danish Twin Registry, *STR* Swedish Twin Registry, *OATS* Older Australian Twins Study, *CAATSA* Carolina African American Twin Study of Aging, *MIDUS* Midlife in the United States, *NASNRC* National Academy of Sciences-National Research Council, *VETSA* Vietnam Era Twin Study of Aging, *LDI* latent dementia index. Dementia is operationalized as clinical diagnoses where available or dementia designated by a cut-off on the LDI score. *N* the number of individuals who contribute to models. Zygosity is each individual’s zygosity. *MZ* monozygotic, *DZ* dizygotic, *SS* same sex pairs, *OS* opposite sex pairs. Education is the mean on the 9-category International Standard Classification of Education (ISCED) code

Age for those with a clinical dementia workup is age at dementia diagnosis or age at last follow-up. For those without a clinical dementia workup, age is age at LDI assessment. M = mean, SD = standard deviation, % = percent

**Table 2:** Results of between-within regression models for education predicting dementia

	Model 1: Phenotypic	Model 2: Between/Within	Model 3: Adjusting for Zygosity	Model 4: Adjusting for Zygosity and Sex
**−2loglikelihood**	321265.6	324298.0	324312.1	325746.1
**DF**	60,018	60,017	60,015	60,010
**Fixed Effects**	B(se)	B(se)	B(se)	B(se)
intercept	−1.27(0.05)****	− 0.81(0.05) ****	− 0.80(0.06) ****	− 0.99(0.06) ****
Educ_ij_ [π]	**− 0.68(0.03)** ****			
Educ_j_ [π_1_]		**−0.98(0.03)** ****	**− 0.98(0.03)** ****	−**0.98(0.03)** ****
Educ_ij_ [π_2_]		− 0.07(0.04)	0.13(0.09)	0.13(0.09)
Zyg_j_ [π_3_]			− 0.02(0.03)	− 0.01(0.03)
Educ_ij_*Zyg_j_ [π_4_]			**− 0.26(0.10)** *	**− 0.27(0.10)** *
Female_ij_ [π_5_]				**0.33(0.03)** ****
Female_ij_*Educ_ij_ [π_6_]				
Female_ij_*Zyg_j_ [π_7_]				
Female_ij_*Zyg_j_*Educ_ij_ [π_8_]				
**Random Effects**				
MZ twin pair	2.54(0.13)	2.34(0.14)	2.30(0.13)	2.09(0.13)
w/in MZ residual	0.80(0.01)	0.84(0.01)	0.84(0.01)	0.87(0.01)
DZ twin pair	1.16(0.07)	1.05(0.07)	1.05(0.07)	0.95(0.07)
w/in DZ residual	0.88(0.01)	0.91(0.01)	0.91(0.01)	0.93(0.01)

N = 60,027 in all models. Dementia is operationalized as clinical diagnoses where available or dementia designated by a cut-off on the latent dementia index (LDI) score. Age (centered at 60 years), Sample, and Sample*Female are included in the model. Zyg = Zygosity (monozygotic, MZ = 0, dizygotic, DZ = 1). Educ = educational attainment using three categories derived from ISCED

*DF* degrees of freedom, *B(se)* change in log-odds of dementia given a unit change in the covariate (standard error)

Significant effects are bolded.

* *p* < 0.05,

**** *p* < 0.0001

## Data Availability

No data were collected specifically for this study, as the study is entirely secondary analysis of deidentified existing data, accessed through study-specific data use agreements with USC. Different members of the IGEMS consortium are governed by different data sharing constraints. Data from several studies are available for download through the National Archive of Computerized Data on Aging (NACDA). Further information is provided on the home pages of the studies’ respective websites. All analysis scripts are available on GitHub.
