# Supplementary material for: Is educational attainment protective against developing dementia? A twin study of genetic and environmental contributions
Source: Eur J Epidemiol. 2025 Aug 12;40(10):1177–90. doi: 10.1007/s10654-025-01286-x (PMC12504008; doi:10.1007/s10654-025-01286-x)
Supplement: Supplementary file 1 — Supplementary Material 1 [file 10654_2025_1286_MOESM1_ESM.pdf]

Supplementary Information for

European Journal of Epidemiology

Is Educational Attainment Protective Against Developing Dementia? A Twin Study of Genetic and Environmental Contributions

Ellen E. Walters[1], Susan E. Luczak[2], Christopher R. Beam[2,3], Malin Ericsson[4], William S. Kremen[5], Robert F. Krueger[6], Kristian E. Markon[6], Matt McGue[6,7], Marianne Nygaard[7], Matthew S. Panizzon[5], Brenda L. Plassman[8], Chandra A. Reynolds[9], Perminder S. Sachdev[10], Anbu Thalamuthu[10], Keith E. Whitfield[11], Nancy L. Pedersen[4], Margaret Gatz[1], for the IGEMS Consortium

[1] University of Southern California, Center for Economic and Social Research, Los Angeles, CA 90089, USA
[2] University of Southern California, Department of Psychology, Los Angeles, CA 90089, USA
[3] University of Southern California, Davis School of Gerontology, Los Angeles, CA 90089, USA
[4] Karolinska Institutet, Department of Medical Epidemiology and Biostatistics, 171 77 Stockholm, Sweden
[5] University of California, San Diego, Department of Psychiatry, La Jolla, CA 92093, USA
[6] University of Minnesota, Department of Psychology, Minneapolis, MN 55455, USA
[7] University of Southern Denmark, The Danish Twin Registry, Department of Public Health, DK-5230 Odense M, Denmark
[8] Duke University, Department of Psychiatry and Behavioral Sciences, Durham, NC 27710, USA
[9] University of Colorado Boulder, Department of Psychology & Neuroscience, Boulder, CO 80309, USA
[10] University of New South Wales, Centre for Healthy Brain Ageing (CHeBA), Sydney, New South Wales 2052, Australia
[11] University of Nevada, Las Vegas, Department of Psychology, Las Vegas, NV 89154, USA

Corresponding Author

Margaret Gatz, University of Southern California, Center for Economic and Social Research, 635 Downey Way, Los Angeles CA 90089-3332. Phone: (213) 740-2212. Email: gatz@usc.edu

Figure S1. Mean difference in educational attainment within pairs, by genetic relationship and sex.

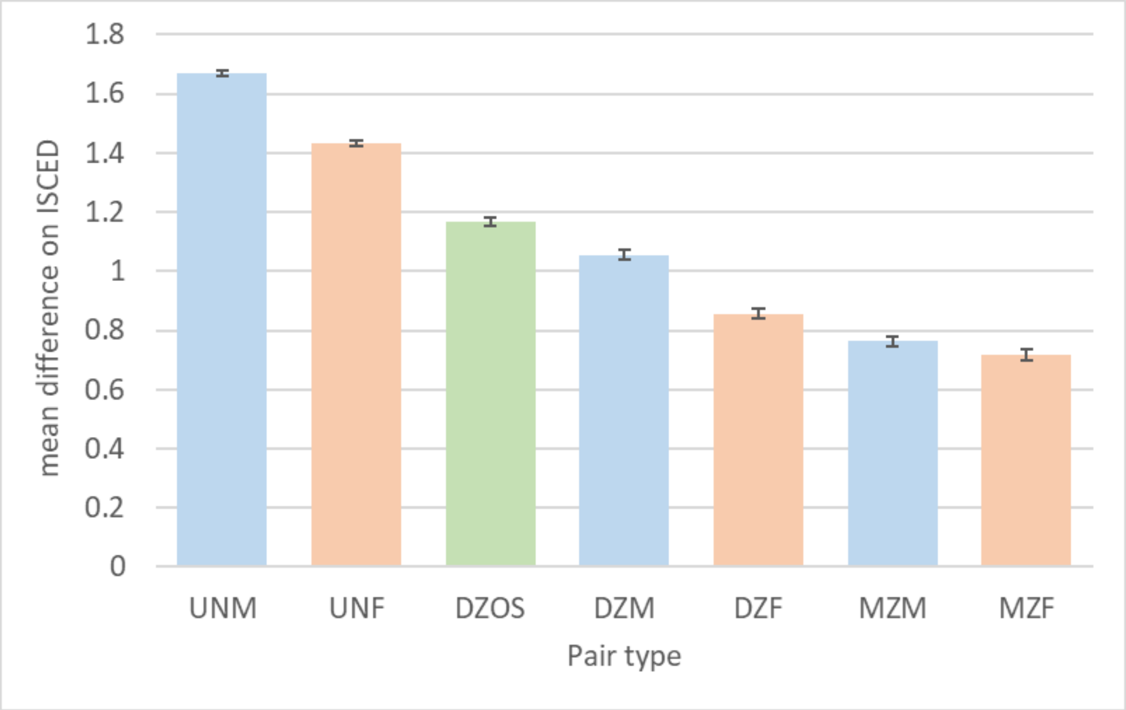

Notes: ISCED= International Standard Classification of Education. UNM = unrelated male matched pairs (N=3437); UNF = unrelated female matched pairs (N=3076); DZOS = opposite sex dizygotic twin pairs (N=551), DZM = male same sex dizygotic twin pairs (N=517); DZF = female same sex dizygotic twin pairs (N=500); MZM = male monozygotic twin pairs (N=520); MZF = female monozygotic twin pairs (N=360). Blue columns indicate male pairs, peach columns indicate female pairs, the green column indicates opposite sex pairs. The bars are standard errors.

Table S1. Results of complete between-within regression models for education predicting dementia

	Model 1: Phenotypic	Model 2: Between/ Within	Model 3: adjusting for zygosity	Model 4: adjusting for zygosity and sex	Model 5: adjusting for zygosity, sex and sex interactions
**2loglikelihood**	321265.6	324298.0	324312.1	325746.1	325799.2
**DF**	60018	60017	60015	60010	60007
**Fixed Effects**	B(se)	B(se)	B(se)	B(se)	B(se)
Intercept	-1.27(.05)*	-0.81(.05)*	-0.80(.06)*	-0.99(.06)*	-0.96(.07)*
$Educ_{ij}$ [π]	-0.68(.03)*				
$Educ_{j}$ [π_1]		-0.98(.03)*	-0.98(.03)*	-0.98(.03)*	0.08(.13)
$Educ_{ij}$ [π_2]		-0.07(.04)	0.13(.09)	0.13(.09)	-0.98(.03)*
Zyg_{j} [π_3]			-0.02(.03)	-0.01(.03)	-0.04(.05)
$Educ_{ij}*Zyg_{j}$ [π_4]			-0.26(.10)*	-0.27(.10)*	-0.12(.15)
$Female_{ij}$ [π_5]				0.33(.03)*	0.28(.06)*
$Female_{ij}*Educ_{ij}$ [π_6]					0.09(.18)
$Female_{ij}*Zyg_{j}$ [π_7]					0.06(.07)
$Female_{ij}*Zyg_{j}*Educ_{ij}$ [π_8]					-0.28.21)
Age_c60	0.02(.002)*	0.01(.002)*	0.01(.002)*	0.01(.002)*	
DTR	-0.14(.06)*	-0.11(.06)*	0.46(.08)*	0.46(.08)*	
MIDUS	0.34(.14)*	0.57(.14)*	1.23(.19)*	1.22(.19)*	
OATS	-0.77(.21)*	-0.62(.21)*	-0.56(.41)	-0.57(.41)	
CAATSA	0.34(.31)	0.24(.31)	0.15(.45)	0.15(.45)	
VETSA	-2.96(.47)*	-2.76(.48)*	-2.57(.48)*	-2.59(.48)*	
NASNRC	-0.31(.06)	-0.08(.06)	0.12(.06)	0.11(.06)	
STR	0.00	0.00	0.00	0.00	
DTR*Female			-1.08(.11)*	-1.07(.11)*	
MIDUS*Female				-1.30(.28)*	-1.29(.28)*
OATS*Female				-0.15(.48)	-0.13(.48)
CAATSA*Female				-0.71(.62)	-0.71(.62)
VETSA*Female				0.00	0.00
NASNRC*Female				0.00	0.00
STR*Female				0.00	0.00

Notes: N=60,027 in all models. DF=degrees of freedom. Educ = educational attainment using three categories derived from the International Standard Classification of Education (ISCED). Zyg = Zygosity (monozygotic, MZ=0, dizygotic, DZ=1). Age_c60 =age centered at 60 years. B(se)= change in log-odds of dementia given a unit change in the covariate (standard error). DTR=Danish Twin Registry; STR=Swedish Twin Registry; OATS=Older Australian Twins Study; CAATSA=Carolina African American Twin Study of Aging; MIDUS=Midlife in the United States; NASNRC= National Academy of Sciences-National Research Council; VETSA=Vietnam Era Twin Study of Aging. *$p<.05$

Table S2. Results of between-within regression models for education predicting dementia in Black sample

	Model 1: Phenotypic	Model 2: Between/Within	Model 3: adjusting for zygosity
**-2loglikelihood**	2061.17	2059.31	2060.19
**DF**	323	322	320
**Fixed Effects**	B(se)	B(se)	B(se)
Intercept	0.94(1.12)	0.53(1.28)	0.66(1.38)
$Educ_{ij}$ [π]	**-2.17(0.64)***		
$Educ_j$ [π_1]		**-1.97(0.70)***	-2.19(0.78)*
$Educ_{ij}$ [π_2]		**-2.76(1.10)***	-2.92(2.13)
Zyg_j [π_3]			0.43(0.69)
$Educ_{ij}$*Zyg_j [π_4]			0.17(2.44)
**Random Effects**			
Intercept	3.90(2.13)	4.21(2.02)	5.96(2.12)
Residual	0.74(0.09)	0.72(0.08)	0.63(0.07)

Notes: N=327 in all models. Dementia is operationalized as clinical diagnoses where available, or cutoff points on the latent dementia indicator (LDI) score. Age (centered at 60 years) and Sample included in the model. Random effects modeled jointly for MZ and DZ twins. Zyg = DF=degrees of freedom. Educ = educational attainment using three categories derived from the International Standard Classification of Education (ISCED). Zyg = Zygosity (monozygotic, MZ=0, dizygotic, DZ=1). B(se)=change in log-odds of dementia given a unit change in the covariate (standard error). *p<.05.

Table S3. Models for education predicting dementia, including potential confounders of dementia risk

	model with ISCED only		model with ISCED and covariate				
	ISCED		ISCED		Covariate		
N	Beta	p-value	Beta	p-value		Beta	p-value
60027	-0.68	<.0001					
52139	-0.62	<.0001	-0.61	<.0001	BMI	0.01	0.0101
50422	-0.62	<.0001	-0.59	<.0001	CIRS score	0.15	<.0001
26318	-0.26	<.0001	-0.26	<.0001	Hypertension	0.06	0.1908
26500	-0.26	<.0001	-0.26	<.0001	Stroke	0.34	<.0001
26540	-0.26	<.0001	-0.26	<.0001	Endocrine disorders	0.05	0.393
15241	-0.14	0.0108	-0.11	0.0541	Physical leisure activities	-0.01	0.0002
16003	-0.16	0.0048	-0.16	0.0070	Social leisure activities	-0.01	0.0394

Note: Dementia is operationalized as clinical diagnoses where available, or cutoff points on the latent dementia indicator (LDI) score. Left panel shows results predicting dementia with educational attainment. Right panel shows same model including the named covariate. The beta for ISCED can be compared in the model without the covariate to the model with the covariate to suggest the importance of the covariate to predicting dementia. LDI=Latent Dementia Index. ISCED= International Standard Classification of Education. BMI=body mass index. CIRS=Cumulation Illness Rating Scale.

Table S4. Twin Pairs Discordant on Dementia and Discordant on Attained Education

	**Case with dementia higher in education**		**Co-twin control higher in education**		**OR (95%CI) in pairs discordant on both dementia and education**	
**Using clinical diagnoses**	**MZ**	**DZ**	**MZ**	**DZ**	**MZ**	**DZ**
STR (pairs discordant for disease)	92 (44%)	431 (48%)	119 (56%)	472 (52%)	1.29 (0.99, 1.70) n=211 pairs	1.10 (0.96, 1.25) n=903 pairs
total	523 (47%)		591 (53%)		1.13 (1.01, 1.27)	
STR (inclusive of pairs discordant for age of onset)	110 (44%)	476 (47%)	136 (56%)	528 (53%)	1.21 (0.94, 1.56) n=241 pairs	1.06 (0.94, 1.20) n=1004 pairs
total	581		664		1.09 (0.97, 1.22)	
NAS-NRC	26 (58%)	41 (49%)	19 (42%)	42 (51%)	0.73 (0.40, 1.32) n=45 pairs	1.02 (0.67, 1.58) n=83 pairs
total	67 (52%)		61 (48%)		0.91 (0.64, 1.29)	
**Using LDI cutoff score**	**MZ**	**DZ**	**MZ**	**DZ**	**MZ**	**DZ**
MIDUS	8 (62%)	6 (46%)	5 (38%)	7 (54%)	0.63 (0.20, 1.91) n=13 pairs	1.17 (0.39, 3.47) n=13 pairs
total	14 (54%)		12 (46%)		0.86 (0.40, 1.85)	
DTR	27 (59%)	34 (28%)	19 (41%)	87 (72%)	0.70 (0.39, 1.27) n=46 pairs	**2.56 (1.72, 3.80) n=121 pairs**
total	61 (37%)		106 (63%)		**1.73 (1.26, 2.36)**	

Notes: Comparison of twin pairs discordant disease, i.e., clinical dementia diagnosis or LDI cut-off score (twin with dementia = case; co-twin without dementia = control, with control twin having reached the age of onset of dementia in the case twin). The analysis was repeated with longitudinal STR data to include twins discordant for age of onset where control twins developed dementia at least 5 years after onset in the case twin. Used conditional logistic regression (survival Cox regression with a case-control dummy variable) to obtain 95% CIs. An OR significantly greater than 1.00 supports the hypothesis that higher education is protective. Pairs concordant for dementia and/or for education not shown. STR=Swedish Twin Registry. NAS-NRC = National Academy of Sciences-National Research Council. MIDUS=Midlife in the United States. DTR=Danish Twin Registry. MZ=monozygotic. DZ=dizygotic. OR=odds ratio. CI=confidence interval. N indicates number of pairs. Bolded results are statistically significant.
